# Implementation of a Real-Time Medication Intake Monitoring Technology Intervention in Community Pharmacy Settings: A Mixed-Method Pilot Study

**DOI:** 10.3390/pharmacy9020105

**Published:** 2021-05-25

**Authors:** Sadaf Faisal, Jessica Ivo, Ryan Tennant, Kelsey-Ann Prior, Kelly Grindrod, Colleen McMillan, Tejal Patel

**Affiliations:** 1 School of Pharmacy, University of Waterloo, Kitchener, ON N2G 1C5, Canada; sadaf.faisal@uwaterloo.ca (S.F.); jarivo@uwaterloo.ca (J.I.); km2prior@uwaterloo.ca (K.-A.P.); kelly.grindrod@uwaterloo.ca (K.G.); 2 System Design Engineering, Faculty of Engineering, University of Waterloo, Waterloo, ON N2L 3G1, Canada; ryan.tennant@uwaterloo.ca; 3 Renison University College, University of Waterloo, Waterloo, ON N2L 3G4, Canada; c7mcmillan@uwaterloo.ca; 4 Research Institute for Aging, Schlegel-University of Waterloo, Waterloo, ON N2J 0E2, Canada

**Keywords:** medication adherence, pharmacists, real-time monitoring, medication dispensing technology

## Abstract

Innovative dispensing products offering real-time medication intake monitoring are being developed to address medication non-adherence. However, implementation of these interventions within the workflow of a community pharmacy is unknown. The purpose of this study was to explore factors affecting implementation of a real-time adherence-monitoring, multidose-dispensing system in community pharmacies. A mixed-method study was conducted with pharmacy staff, who packaged and dispensed medications in smart multidose packages and monitored real-time medication intake via web-portal. Pharmacy staff participated in semi-structured interviews. The Technology Acceptance Model, Theory of Planned Behaviour and Capability, Opportunity, Motivation, Behaviour Model informed the interview guide. Interview transcripts were analyzed thematically and findings were mapped back to the frameworks. The usability was assessed by the System Usability Scale (SUS). Three pharmacists and one pharmacy assistant with a mean of 19 years of practice were interviewed. Three themes and 12 subthemes were generated. Themes included: pharmacy workflow factors, integration factors, and pharmacist-perceived patient factors. The mean SUS was found to be 80.63. Products with real-time adherence monitoring capabilities are valued by pharmacists. A careful assessment of infrastructure—including pharmacy workload, manpower and financial resources—is imperative for successful implementation of such interventions in a community pharmacy setting.

## 1. Introduction

Non-adherence to therapies is a global healthcare challenge. In developed countries, medication adherence is reported to be approximately 50% in patients with chronic illnesses [1]. Medication non-adherence has been linked to negative health outcomes for patients, as well as increased costs to healthcare systems. For example, a population-based cohort study in patients with hypertension reported a higher risk of stroke (1.13 and 1.27 times respectively) with intermediate and poor adherence to antihypertensive medication, as compared to those with high adherence [2]. Another study examining the effect of medication non-adherence on healthcare costs in diabetic patients demonstrated that improving adherence can save approximately $661 million to $1.16 billion annually [3]. Assessing adherence is important, not only to determine the extent of non-adherence, but also to identify factors and patterns of non-adherence [4,5]. Medication adherence can be determined directly by measuring drug or metabolite levels in the bodily fluids or indirectly by assessing prescription records, pill counts, patient self-reports through interviews, questionnaires or diaries, and/or electronic medication packaging devices [5].

Obtaining adherence data electronically can be a useful way to provide patients with feedback to improve non-adherent behaviour [6]. The data obtained through this method document the date and time the medication was accessed [7]. Several studies have reported on the impact of electronic medication adherence feedback on medication adherence, clinical outcomes and hospitalization [6,8]. A systematic review assessing the effect of electronic adherence monitoring feedback on adherence and clinical outcomes reported a positive impact on adherence [6]. However, in this systematic review, the impact of electronic monitoring and feedback on clinical outcomes was found to be inconclusive. In another randomized controlled study, electronic monitoring and feedback significantly decreased the need for oral steroids (*p* = 0.008) and hospital admissions (*p* ≤ 0.0010) in the active arm compared to the control group among pediatric asthma patients [8]. Real-time medication intake monitoring is an innovative approach of adherence monitoring. It offers healthcare providers a unique opportunity to monitor patients for their medication intake and intervene in a timely manner.

Healthcare providers, especially pharmacists, are in an ideal position to address and assist patients in improving adherence. Pharmacists are highly accessible and trusted professionals with expertise in medication management [9]. Community pharmacists see their patients face-to-face regularly, which provides opportunities for building relationships and communicating directly with patients [10]. A recent study in Canada reported that approximately 55% of Canadians visit their community pharmacy at least once weekly [11]. Numerous interventions are offered by pharmacists to improve medication adherence for their patients. These interventions range from patient education and counselling, simplifying dosage regimens, packaging medications for convenient administration, conducting medication reviews and many more [12,13,14,15]. Studies indicate that these interventions impact adherence in a positive manner. For example, a systematic review examining the impact of community pharmacist-led interventions on medication adherence and health outcomes reported that improvement in adherence resulted in improvements in blood pressure, cholesterol, asthma, and chronic obstructive pulmonary disease control [16]. Another randomized controlled study evaluating the impact of community pharmacist-led adherence interventions on adherence, healthcare utilization, and costs showed that the intervention group reported 3% higher medication adherence, 1.8% fewer hospital admissions, and 2.7% less emergency room visits as compared to the control group [17]. In yet another randomized controlled trial assessing the impact of pharmacist intervention on adherence in low-income heart failure patients, the intervention improved adherence to 78.8%, compared to 67.9% in the usual care group. The improvement in adherence resulted in 19.4% fewer emergency room visits and hospital admissions and reduced annual healthcare costs. Of note, medication adherence was measured through the use of Medication Event Monitoring System (MEMS) prescription container lids, which enable monitoring of medication intake [18].

Innovative medication-based technologies, such as automated dispensers, electronic dosettes or pill boxes, electronic blister packs, electronic inhaler devices and electronic injectors have been developing over the years [7,19,20]. This growth in development has led to an abundance of adherence products capable of real-time adherence monitoring on the market for in-home patient use [7,19,20]. These adherence products have a variety of features; however, their most notable feature is the ability to send notifications and reminders to the patient and/or caregivers when a dose is due to be ingested. Some of these products require pharmacies to package and dispense the medications, while other products require that the caregiver or patient fill the device with medication doses [21,22,23].

The impact of packaging and dispensing medications to meet the requirements of these new technologies and the feasibility of monitoring real-time medication intake within community pharmacy settings is not known. Several barriers and facilitators related to implementation of clinical services and programs in pharmacies have been identified [24,25,26,27]. It is necessary to identify and understand factors that can enable or hinder the successful implementation of an innovative clinical service at the pharmacy level.

The usability of a product can be defined as “the extent to which a product can be used by a specified user” [28]. For the successful adoption of any innovative system, usability should be determined to identify problems with the product design or how easy or difficult the product is to use, as these factors can drive the intention to use the product. Therefore, we conducted a pilot study using mixed methods, with community pharmacies dispensing a prototype smart medication adherence product with the capability of real-time medication intake monitoring. The purpose of this study was to understand the factors which may impact community pharmacies offering these products to their non-adherent patients and explore the usability of a prototype smart adherence technology system.

## 2. Materials and Methods

### 2.1. Theoretical Framework

Three validated frameworks were used to inform this research: (1) the Technology Acceptance Model (TAM), (2) Theory of Planned Behaviour (TPB), and (3) Capability, Opportunity, Motivation, Behaviour (COM-B) Model. It has been argued that TAM alone cannot predict healthcare providers’ beliefs about the use of health-related technology [29], therefore, we used an integrated approach of combing two behaviour theories—TPB and COM-B Model with TAM framework—to explore the factors affecting the implementation of technology-based adherence intervention in a community pharmacy setting, and to add rigour to the study.

The TAM framework is a validated framework frequently used in pharmacy research to assess a user’s acceptance and intention to adopt a technology [30,31,32]. The framework suggests that a user’s actual use of a technology depends on their intention to adopt the technology. This intention to use a technology can be based on a user’s perceived usefulness and perceived ease of use, along with other external factors [30]. The TPB is one of several health behaviour theories which posit that an individual’s engagement in a behaviour can be influenced by their intention, their own beliefs about the behaviour, others’ attitudes toward the behaviour, and factors that can facilitate or impede the behaviour [33]. The COM-B model is a framework to identify and understand the factors that can affect a behaviour change [34]. This model states that any behaviour is dependent on the capability of an individual to perform a behaviour and available opportunities and motivation to engage in the behaviour. Both TPB and COM-B Model have been used in pharmacy research to understand and predict behaviour [35,36,37,38,39,40].

### 2.2. System Usability Scale

The system usability scale (SUS) is a validated tool used to measure the usability of a product [41]. The tool has been used to assess the usability of, cell phones, appliances such as TV and microwaves and websites [42]. Recently, SUS has been utilized in healthcare to evaluate the usability of internet-based healthcare interventions used by professionals, electronic health records, home healthcare devices, mobile health applications, and electronic medication adherence products [43,44,45,46,47].

### 2.3. Study Design

A mixed-method study design was used for this study. This study was part of a larger ethnographic-informed study focusing on the integration of a smart multidose blister package for in-home medication management in older adults with chronic disease.

### 2.4. Study Material

#### Smart Adherence Technology System

**Figure 1:**
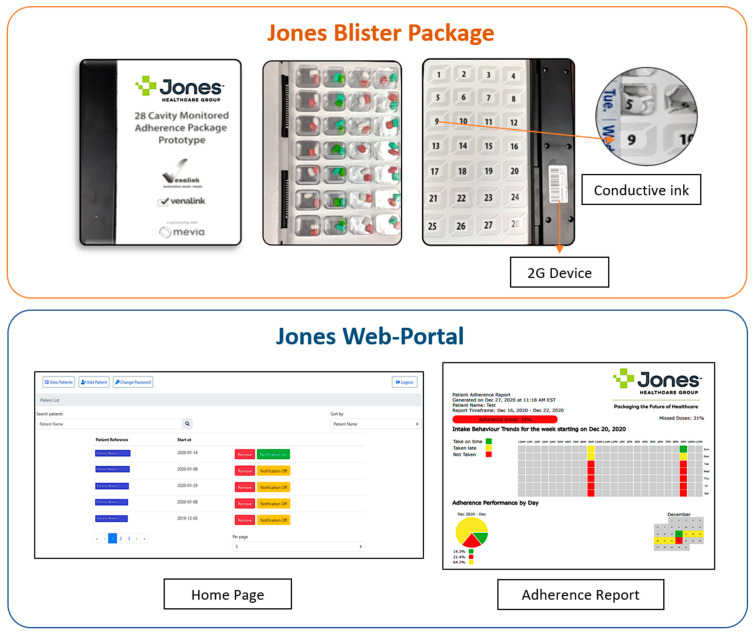
Description of Adherence Technology System.

The smart adherence technology system was comprised of a smart multidose blister package and a web-based portal to monitor the patient’s medication intake remotely (see Figure 1 for a description of the adherence technology system).

(a) Smart multidose blister package

The smart multidose blister package is a prototype product using telecommunication technology. The blister package consists of a plastic blister, aluminum foil substrate, and a paperboard with conductive ink circuitry that enables recording of dosage events. The blister pack is comprised of 28 cavities and provides up to four times dosing of multiple medications for the duration of one week. A telecommunications device is attached to the individual disposable adherence blister package. The package is pre-filled by the pharmacy. When a cavity is broken to access the medications, the telecommunications device records the medication intake event and uploads the data to a cloud-based software portal. The system generates reminders and notifications via global system for mobile communications (GSM) and short message service (SMS) technologies, which are sent as text to a mobile phone or an email address.

(b) Cloud-based Software Portal

**Figure 2:**
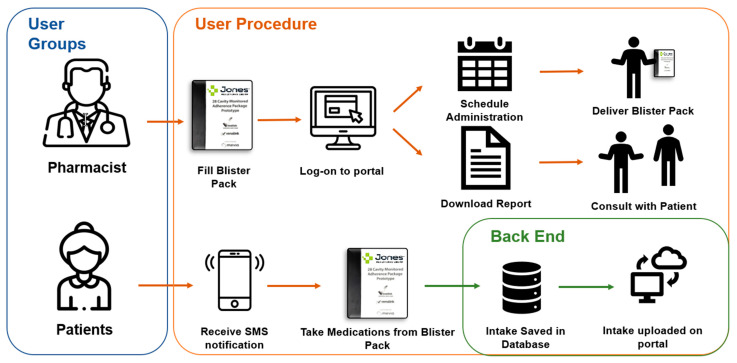
Smart Adherence Technology System Process.

The software portal is the online interface that can be accessed by a healthcare professional. The portal can be used to set patient medication schedules, set up notifications and obtain a report on patient medication adherence. The portal displays all information transmitted by the telecommunications device and includes a summary page displaying events for all patient attached to a user’s account. Additionally, each account has a patient profile page providing patient information, device, battery status, service connection status and date range for monitoring (see Figure 2 for the process of the smart adherence technology system).

### 2.5. Ethical Consideration

The study received ethics approval from the University of Waterloo Clinical Research Ethics Committee (ORE # 41015), Canada. All participants provided written consent prior to the start of study.

The study was conducted in three community pharmacies located in two major cities in Ontario, Canada between November 2019 to June 2020.

### 2.6. Study Participants

Pharmacists and pharmacy assistants who packaged and dispensed medications in smart multidose packages and monitored real-time medication intake via a web-portal remotely for older adults were recruited. Recruitment flyers, patient participants and professional networks of researchers were used to recruit the pharmacies.

### 2.7. Study Procedures

Once older adult participants provided permission to contact their respective pharmacies, information letters and consent forms were sent to the pharmacy staff. Pharmacists and pharmacy assistants were provided with in-person training on how to set up and use the smart adherence system, including the physical device and web-based portal. Training was provided over 45 to 60 min. Following the training session, community pharmacies packaged and dispensed each participant’s regular medications in the smart blister package for eight weeks and monitored real time medication intake remotely. Each patient was assigned with three telecommunication devices to be attached to each individual weekly disposable blister package. At the end of the study, pharmacy staff were asked to participate in a one-on-one interview and complete the System Usability Scale (SUS).

### 2.8. Data Collection

#### 2.8.1. Qualitative: Semi-structured Interviews

At the end of the study period, pharmacy staff were asked to partake in one-on-one semi-structured interviews to provide their feedback and experience related to the implementation of the smart adherence system. The interview guide was developed based on the constructs of the TAM, TPB, and COM-B models. The guide was initially developed by two researchers (SF and JI) with backgrounds in pharmacy and health informatics. One other researcher (TP), with pharmacy practice research experience, reviewed the interview guide prior to finalizing it through discussion and agreement (see Supplementary Table S1 for the complete interview guide). Three team members (JI, RT and SF) conducted the interviews. Each interview lasted from 60 to 90 min. Due to the coronavirus disease 2019 (COVID-19) pandemic declaration, all interviews were conducted virtually via telephone or video conferencing software. All interviews were audio recorded by using Sony IC recorder ICD-PX470 and transcribed verbatim by four team members (A.S., D.J., K.P. and A.P.) using Microsoft Word (Microsoft^®^ for Mac version 16.16.13). One of the four team members reviewed the transcripts for accuracy. Interview transcripts were entered into NVivo 11 software (QSR international, Melbourne, Australia) to manage and analyze data.

#### 2.8.2. Quantitative: System Usability Scale

At the end of the study, all participants were asked to complete the SUS questionnaire. The SUS questionnaire was comprised of 10 questions on a five-point Likert scale ranging from “Strongly Disagree” to “Strongly Agree”. The scores ranged from 0 to 100, with a higher score indicating that the product was more usable.

### 2.9. Data Analysis

Braun and Clark’s framework (2006) of thematic analysis was used to analyze the interviews [48]. Two team members (S.F. and J.I.) read and reread the interviews to become familiar with data. NVivo 11 (QSR international, Melbourne, Australia) was used to organize and code the data. Both team members coded the first interview independently, then came together to discuss the codes to ensure the consistency of codes and establish the code book. All three remaining interviews were coded independently by both researchers (J.I. and S.F.) based on the established code book. To add rigour to the study, we also calculated the inter-rater reliability between the two coders, which is a benchmark of qualitative studies. The inter-rater reliability was found to be 88.6%. Finally, to add even more rigour, four researchers (K.P., J.I., R.T., S.F.) reviewed the code book together. Any disagreements were resolved by discussion. Codes were grouped into themes and sub-themes. Once themes were finalized, four researchers (K.P., J.I., S.F. and R.T.) reviewed them once again. Since the frameworks were guiding the research, themes and sub-themes were mapped back to the TAM, TPB and COM-B Model to understand the meanings of results.

Member checking was performed to ensure the trustworthiness of the data. Member checking is a process of asking participants to review the research findings and confirm the validity of the data [49]. A document containing summarized details of the themes, sub-themes and de-identified participant quotations were sent to all participants via e-mail. Fifty percent of study participants responded to our member checking process and agreed with the interpretation of the results. No disagreements were noted by participants and as such, no changes were made to the original themes and sub-themes.

## 3. Results

### 3.1. Demographic Characteristics

Five participants were recruited; however, one participant was lost to follow-up and did not participate in the final one-on-one interview. Three pharmacists and one pharmacy assistant participated in the one-on-one semi-structured interview. All participants recommended medication adherence aid(s) to their patients (see Table 1).

### 3.2. Themes and Sub-Themes

Our interview analysis identified three themes and 12 sub-themes, which are described below without any hierarchy related to their importance.

#### 3.2.1. Theme 1: Pharmacy Workflow Related Factors

##### Sub-Theme 1.1: Pharmacy Workload

Pharmacy workload (due to added steps involved in packaging the medications in smart blister package and entering patient data on the web-portal) was one sub-theme identified by the interview analysis. Participants compared the workload involved in adopting this system to their usual system of pharmacy prepared regular blister packs.

“You have to assign the devices to every patient, you have to make sure that the devices are charged and ready to go. You have to keep an inventory of the devices to make sure that they’re going and coming back and the patient is not just keeping them at home. So... there’s more work involved around the managing of this whole system as a smart blister pack for the pharmacy team”-004HCP.

Participants also discussed the perceived workload if they decided to implement the system for all of their patients.

“Right now, I have ninety patients on blister packs. If I were to add fifteen minutes every week for ninety patients, you can imagine how much extra time it would take and that’s not taking the conversations that will happen because I am seeing the compliance reports. So that in itself is a huge undertaking for any pharmacy”-004HCP.

##### Sub-Theme 1.2: Staff Availability, Training and Role

When asked if there were any resources needed to adopt the system in their pharmacies, participants stated that additional staff or manpower would be required to maintain this system adequately to not only fill the blister package, but also enter and regularly review data from the portal. 

“Let’s say 10 or 15 patients…just from an actual delivery and keeping track of all those things… that would be a little bit tricky from a…gain, more staff time needed that’s all” -005HCP.

In addition to manpower, staff training was indicated as another factor. Participants mentioned that due to the novelty of this system, pharmacy staff would require additional training. Some of the pharmacy staff showed hesitation in filling the smart blister packages, despite the fact that they were filling regular blister packs which looked exactly similar to the smart blister package but without the paperboard with conductive ink circuitry and connectivity device for non-study patients. In other cases, some pharmacists did not feel comfortable involving the pharmacy staff in the process of preparing smart blister package.

“Although my assistant would have done it…I wasn’t very comfortable in, in letting her do it” -004HCP.

Another important aspect identified during the interview analysis was the role differentiation among healthcare providers in the pharmacy.

“For filling we had a pharmacy assistant who was filling the blister pack. Other than that, everything else was done by a pharmacist. So, printing it…keeping you know…the schedule that…you know, they have to be printed then they have to be made. If there’s any changes…with the setting it up initially and then copying it over for the next week, all of those things were done by a pharmacist in the pharmacy. So, the only thing that was left for the pharmacy assistant to do was just with filling of the blister packs”-004HCP.

##### Sub-Theme 1.3: Pharmacy Workflow Organization

Participants mentioned that they needed to make some changes in their pharmacy workflow, which involved dedicating a specific place for blister pack storage, charging the batteries and preparing the blister packs.

“You would want to make sure that you have a dedicated area to keep track of the charge units, what’s charged, [and] what’s not. Because of…packaging…is different from the other packaging that we have at the store, it needed its own little area as well so maybe some space in that, you know some planning around that”-005HCP.

They also mentioned that additional support was required from delivery staff to collect the old blister packs and deliver the new ones. This also led to multiple trips to patients’ homes and required planning.

“We just have to do those little nuances to figure out that system ahead of time and maybe it means that family has to drop...that box off in the middle of the week to the pharmacy and it’s not delivered”-004HCP.

##### Sub-Theme 1.4: Cost Associated to Set up the System

Due to additional staff, workload and time requirement for system implementation, associated cost was mentioned as an important factor.

“At this point it would have to be […] economically viable [be] because it does take more time on a pharmacy stand point just you know- even from the delivery stand point”-005HCP.

Pharmacists also mentioned additional remuneration for added workload when monitoring the real-time medication intake remotely.

“If you didn’t have some sort of remuneration system I don’t see any…advantage to a pharmacy to actually take this on unless they are getting paid more. Just from a time standpoint again, it only takes a couple minutes to set up a card, no big deal, but if I’m gonna start going back and analyzing it, that’s just going to take a lot more time”-005HCP.

##### Sub-Theme 1.5: Regulatory Implication

Pharmacists stated that having the ability to monitor real-time medication intake put them in a position where they needed to know about the regulatory implications of this data.

“You have to think about the pharmacist, the position that this data will put the pharmacist in. You cannot, if you have a printed report in front of you, and of course we want to have that printed report as you want to see what the compliance is, but you cannot then to choose to ignore that report if there’s a compliance issue. Because that would put the pharmacist in a very bad position legally, that they had printed report in front of them that the patient was not complying, and they failed to act on that. Right now, we don’t have that information, so we don’t know”-004HCP.

##### Sub-Theme 1.6: Feedback from Others

Participants mentioned they received variable feedback from others around, including pharmacy staff and patients with the technology. Pharmacy staff who were not involved in the study commented on the additional workload and did not want to participate.

“I think the only comments they made was thinking that it was a lot of work because I was trimming all the stickers, I had to cut both sides and the top and bottom, so that took a while they saw me working on that”-018HCP.

When asked about the response from the physicians, participants thought they would embrace this technology as it will help their patients to adhere to medications.

“Absolutely, yeah absolutely, I think. Physicians you know, they love it that the medications that they’re prescribing, the patients are taking those medications and they’re compliant…I think there’s nothing more they love than that, you know, they prescribe a prescription medication and the patient doesn’t take it for two months they go back and see the physician and the physician thinks that they’re medications are not working and they increase the dose and they keep doing it and the patient’s just not taking the medication. So, for them to know exactly what’s going on I think it can help them quite a bit in…treating their patient. So, I think the physician would be very receptive”-004HCP.

##### Sub-Theme 1.7: Improved Patient Interaction

Participants mentioned that they felt their interactions with their patients were improved due to the system.

“People were happy that we had called them, they were interested in being involved in a study, they were interested in being involved in something new”-005HCP.

#### 3.2.2. Theme 2: Integration Related Factors

##### Sub-Theme 2.1: Product Design Factors

When asked about their experience related to dispensing medications using the smart blister package, participants felt that the system was easy to implement and they did not experience any issues while dispensing the medications in the blister package. However, the size and bulkiness of the blister package was not appreciated by both pharmacy staff and the patients.

“One thing was that I think most of the patients found it to be quite…you know they have to keep it somewhere because of the device it’s, you know, and for us to, to store it, to keep it, send it, it’s always a bulkier item to send”-004HCP.

Since each patient was assigned with three telecommunication devices thus the limited availability of these devices created some logistics issue for the pharmacy. Pharmacy delivery staff had to make extra trips to patients’ homes to pick up the devices. Some patients prefer to have their medications on monthly basis rather than weekly basis and due to limited quantity of devices, it was not possible for the pharmacy to deliver four blister packs.

“I think considering we had the two weeks supply…you’re gonna need more of those black boxes because some of the patients would go to monthly”-005HCP.

##### Sub-Theme 2.2: Portal Factors

Difficulty in setting up patient profiles on the software portal were identified as a problem by all participants. The initial portal set up included adding patient information, setting up the text message reminder and creating a dosing schedule. Pharmacists were also required to create a dosing schedule every week which could be manually completed or cloned from the previous week.

“I don’t think you need any extra special skills, I think um as, as a pharmacist you’re always um dealing with software in pharmacies, so it’s just a, just a maybe a quick overview of software”-004HCP.

However, once the initial set-up was done, participants found the system easy to use.

The access of real-time medication intake data was found to be useful by all the participants. Pharmacists found that this feature could be very useful in addressing medication non-adherence while conducting medication reviews, or in cases in which the family members or caregivers expressed any concerns related to non-adherence of their patients.

“The benefits of it…you know uh not just for that patients, there’s the pharmacist, the healthcare provider can see the compliance, the patient can see the compliance, their family members can see the compliance so there’s, there’s definitely benefit”-004HCP.

Participants faced a few challenges regarding the reliability of the real-time medication intake data. The system experienced some technical difficulties during the study period and did not report the adherence accurately. This was identified as a huge concern by the participants.

“My concern is…about the software…if I can trust the software a 100% and if I know that the software is working 100% is what I’m seeing of the software uh that the compliance is not 100%. If I’m, if I trust the software 100% then having that conversation with the, with the patient is definitely not the problem, I have that conversation all the time with for people who are on regular dosette when they have not been taking their medications” -004HCP.

The portal had the ability to show the adherence percentage as well as show the adherence record in different colours to differentiate if a participant is adherent, or non-adherent. Pharmacists found this feature quite useful.

“It’s a you know, the colour coding when they have not taken their medication on time or when they have taken their medications on time. I think that’s kind of gives you right at one glance you can see the compliance. If the compliance is not there on a person’s page you can just see that they did not take most of their medications on time. That’s one thing…I mean it gives you that- that snapshot of the patient. Uh, it gives you that percentage compliance as well as you know what’s going on”-004HCP.

Overall, participants expressed their satisfaction with the system and commented on their intention to use it in the future.

“If it was available we would offer it”-007HCP.

“I actually enjoyed it I was very satisfied with it”-005HCP.

#### 3.2.3. Theme 3: Pharmacist Perceived Patient Related Factors

##### Sub-Theme 3.1: Potential Users

Pharmacists identified that not only patients, but also family members, could be potential users of this system. They mentioned that such a system could offer people an opportunity for independent living.

“If you have your mom or dad or grandparents living by themselves in a retirement home but they’re still independent, or if they’re living at home [and] they’re taking their own medications… you are not going to go see them every day or maybe not maybe even every week”-004HCP.

##### Sub-Theme 3.2: Concerns for Users

Pharmacists also expressed concerns for some users with cognitive and physical deficits to use the product appropriately. The system sends text reminders to the patients at their scheduled dosing time; however, pharmacists felt that people with advanced stages of cognitive impairment may not be able to process that information and would not be able to respond to the reminder function. Pharmacists identified that, for such situations, it would be beneficial to involve family members.

“We had patients on blisters for a reason because usually some sort of cognitive decline so the question is: is the notification to a patient’s cellphone, is that going to be enough to make a difference or does the notification have to go somewhere else, having a family member involved. Where does the bang for our buck come in do we have a better bang by just have a patient on a smart blister getting notified themselves? You know what if they are with it, then maybe that’s what they want”-005 HCP.

Another concern identified by pharmacists was the ability of patients with physical challenges to access medications from the blister package. The blister package requires a certain process to punch the medications out and it may not be feasible for such patients.

“It was hard to punch for some patients especially with some arthritis or if they have, they have Parkinson’s or their hands are shaking”-004 HCP.

##### Sub-Theme 3.3: Cost to the End-Users

Cost associated with the system’s use was another important concern expressed by pharmacists prior to offering this product to the patients. However, pharmacists felt that the system was still an affordable option for patients, compared to the cost of nurse-led medication administration services for those who wish to live independently in their homes.

“You know, for the whole package including the connectivity fees, [and] pharmacy fees… [would be] less than what they would pay a nurse to come in and give the medications”-004HCP.

##### Sub-Theme 3.4: Technology Access for the End-User

Another important patient factor that was identified by participants was the availability and access of the technology required for the functionality of the smart blister package. The blister package requires a cell phone with the capability of receiving SMS text messages for the reminder function. Participants mentioned that most of the older adults in their practice did not carry a cellphone. Even older adults who did had limited data plans, which were not feasibly able to accommodate the messaging services.

“Well with our two patients’…I don’t know if we had a good sample size, but I don’t think…they had the technology…to fully use the device”-007HCP.

### 3.3. Themes and Sub-Themes Mapped to Theoretical Frameworks

**Figure 3:**
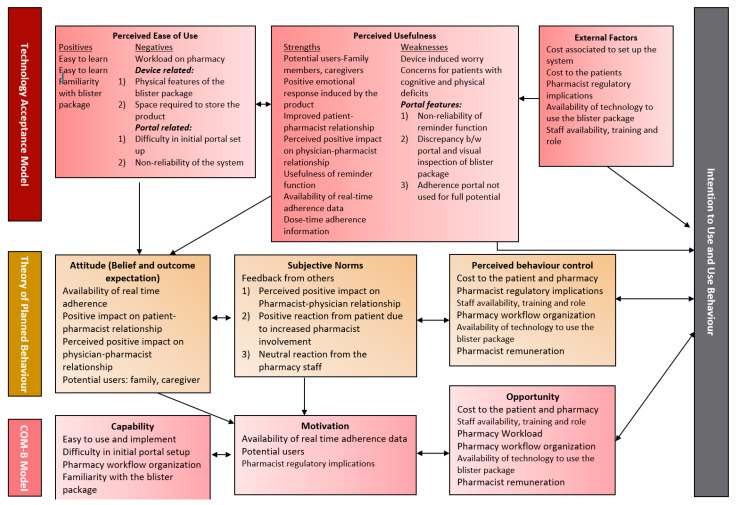
Themes and sub-themes mapped to theoretical frameworks.

The identified themes and sub-themes were mapped back to the theoretical frameworks (see Figure 3).

### 3.4. System Usability Scale

The mean SUS score was found to be 80.63 with a range of 70–87.5.

## 4. Discussion

### 4.1. Principal Findings

To the best of our knowledge, this is the first study to examine the factors that may impact the implementation of a prototype smart adherence system in community pharmacies in Ontario, Canada. Our study results indicated that pharmacists valued the availability of real-time medication intake data and perceived that it could be a useful tool to aid in clinical decision-making related to therapy. We identified numerous factors that were not solely related to pharmacy workflow; they included pharmacists’ perceived patient determinants and product features which may impact the implementation of such interventions in community pharmacy settings.

Previously, studies have identified pharmacy workflow and time constraints as critical barriers to offering medication management interventions at community pharmacies [50,51,52,53]. A systematic review of pharmacy clinical care services identified pharmacy workflow and space availability, time constraint, low remuneration cost and extensive paperwork as potential barriers for pharmacists to adopt clinical services [54]. The interview analysis for this study provided additional and new insights from pharmacists and pharmacy assistants regarding pharmacy workflow related factors. Although participants perceived that the system was easy to use, the initial set up and staff training was noted as a necessary step to implement these services effectively. Thus, it was perceived as a barrier. The additional required steps within the traditional pharmacy workflow organization included switching between the portal screen and the pharmacy software screen, securing the blister packages’ connectivity devices, labelling the blister package’s using date and time stickers, and additional delivery scheduling. Furthermore, pharmacy staff required a larger space and specific storage locations for the smart blister package as compared to traditional blister packs. The pharmacies in this study were dispensing smart blister packages for a small number of patients (one pharmacy dispensed for 5 patients, one for three and one for two patients), and storage of the devices was already a concern. If a pharmacy chose to use this service for dispensation to a large number of patients, it would be important to support those pharmacies in efforts to identify effective methods for storage in order to maintain their current workflow. Moreover, to fully implement the service, pharmacies might require additional staffing or personnel resources. Personnel requirements could include more pharmacy assistants to package the blister packs, pharmacists to monitor and address real-time adherence intake, and personnel responsible for delivering the packages to the patients who cannot commute to the pharmacy. The cost associated with increased staffing, training, and workload must be considered before offering such services.

In our study, pharmacists perceived the usefulness of access to real-time adherence data. They expressed that this access would provide them with the confidence to address non-adherence issues with their patients and positively influence physician-pharmacist interaction. However, given all of the new data available to them, this also placed a regulatory implication on pharmacists. Pharmacists were concerned about balancing the constraints of implementing this system with the time required to identify adherence issues and patterns to intervene if needed effectively. Additionally, pharmacists expressed that there should be reasonable remuneration for the additional time and resources needed to monitor the real-time adherence and follow up with the patients and physicians. Several studies have identified lack of financial remuneration or poor remuneration as a barrier for pharmacists potentially offering clinical services in healthcare systems [55,56,57]. Canada has a universal healthcare funding model [58]. In Ontario, the Ontario Drug Benefit (ODB) program covers the cost of most prescription medications, a few over the counter medications, and some monitoring devices (such as glucometers for specific age groups and populations) [59]. Currently, this program reimburses pharmacies for identifying a potential drug-related problem while dispensing, conducting medication reviews, and supporting smoking cessation for their patients [60]. However, they do not provide reimbursement for pharmacists or pharmacies that offer real-time medication monitoring services. Similarly, none of the private insurance plans provide reimbursement for these kinds of clinical services. If pharmacists were to monitor real-time medication intake for their patients and intervene in a timely manner, this may lead to prevention of hospitalization, emergency room visits and potential healthcare cost savings related to non-adherence. However, currently, pharmacists would not be remunerated for their efforts. Therefore, policy makers should analyze the current pharmacy funding model in both public and private sectors for the successful implementation of such services.

By using the integrated model with three different frameworks, we also identified critical patient and product determinants that were perceived by pharmacists to be related to the safe and effective use of such products. These determinants could help pharmacists identify and recommend the appropriateness of an adherence intervention for their patient population. Medication adherence interventions should be individualized based on patient characteristics [61]. In our study, pharmacists perceived that the smart adherence product could be usable in patients with unintentional non-adherence. However, due to the smart blister package’s physical features, pharmacists indicated that the product might not be suitable for all users, especially those experiencing dexterity issues related to aging or disease state. In those cases, family caregivers were identified as potential users of the system, especially if they live in a different geographical location than their loved one. The prototype smart adherence system provides an opportunity for caregivers to receive notifications about missed doses. The reminder functionality is a valuable feature and can be utilized to address non-adherence promptly.

The cost to the patients and patient access to technology were some of the barriers identified by the pharmacists. The smart blister package requires a cell phone with the ability to receive SMS reminders. Similarly, this system’s costs may include monthly connectivity fees and expenses associated with a more capable cell phone plan. Most of the pharmacies in Ontario, Canada, offer blister packaging as a free service, while some may charge a minimal monthly fee. In this study, the pharmacists raised concerns with costs associated with cell phone plans, as most of their patients either had a basic cell phone or had a cell phone with a limited plan that had a cap on messaging services.

During this study, both patients and pharmacists encountered some technical challenges with the portal and its reminder functionality. This induced panic and worried both patients and pharmacists, who feared that they were doing something wrong with regard to the system. As mentioned above, product features and design can affect a product’s usability in specific patient populations. This could very well impact the integration of such devices into patient’s homes. Therefore, pharmacists should carefully evaluate patient and device-related factors to match them appropriately before offering these types of interventions.

### 4.2. System Usability Scale

The SUS scale is a validated tool to assess the usability of a product subjectively [41]. There is no cut off value available to indicate the usability of a product; however, Bangor et al. interpreted the SUS score by using adjectives. SUS scores higher than 70 indicate that the product is acceptable by the users, while scores between 50–69 indicate that the product is marginally acceptable and scores lower than 50 demonstrate that it is not acceptable [42]. The SUS score for the smart adherence system in this study was reported to be 80.63 with a range of 70 to 87.5. This score indicates that the product was rated acceptable to use by pharmacy staff.

### 4.3. Strengths and Limitations

This is the first study that specifically explored and outlined the implementation of smart adherence interventions at the community pharmacy in Canada (to the best of our knowledge). The study adds to the existing literature related to the barriers and facilitators affecting adherence interventions at community pharmacies. We used three frameworks to develop the interview guide and analyze the data, which provides rigour to the study. A limitation of the study was the small sample size. Although the research was conducted in three community pharmacies, we could only interview three pharmacists and one pharmacy assistant.

## 5. Conclusions

Products that offer real time medication intake monitoring are valued by healthcare providers, especially pharmacists. However, essential determinants related to pharmacy workflow—along with patients and product factors—must be considered before implementing a technology-based adherence intervention program in a community pharmacy setting. The careful evaluation of these factors will help pharmacy teams and management to continuously integrate these services successfully. Future studies should be designed with larger sample sizes and structured as randomized controlled trials that compare healthcare systems’ cost-savings due to the delivery of such adherence interventions via community pharmacies.

## Figures and Tables

**Table 1:** Demographic characteristics of participants.

Variable	(*N* = 4)
Gender (*n*, %) Male	3 (75.00%)
Age (years) Mean ± SD/Range	43 ± 7.9/30–50
Years of Practice Mean ± SD/Range	19 ± 9.7/5–3

## Data Availability

The data presented in this study are available on request from the corresponding author.

## Supplementary Materials

The following are available online at https://www.mdpi.com/article/10.3390/pharmacy9020105/s1, Table S1: Interview Guide.
