# Supplementary material for: Implementation of a Real-Time Medication Intake Monitoring Technology Intervention in Community Pharmacy Settings: A Mixed-Method Pilot Study
Source: Pharmacy (Basel). 2021 May 25;9(2):105. doi: 10.3390/pharmacy9020105 (PMC8162977; doi:10.3390/pharmacy9020105)
Supplement: Supplementary file 1 [file pharmacy-09-00105-s001.zip › pharmacy-1217892-supplementary.pdf]

Supplementary Materials:

Table S1: Interview Guide

**Questions**	**Constructs**	**Framework**
What do you see as advantages/disadvantages of using the smart blister pack by your patient?<br>What did you hear back from your patient regarding the bister pack?<br>What did you see as advantage/disadvantage of access to medication adherence information for your patients	Attitude	Technology Acceptance Model
How did the use of smart blister pack affect your interaction with your patients?<br>What did people around you (other pharmacy staff) think about the smart blister pack?	Subjective Norm	
What skills do you think you need to dispense the smart blister pack or access the portal?<br>How would you explain any planning that was required to use the system?	Perceived behaviour control-Skills	
What type of resources would you need to adopt this system in your pharmacy?	Perceived behaviour control-Resources	
How would you explain your ability to use the system in your pharmacy?<br>What skills do you think you need to dispense the smart blister pack or access the portal?	Capability- Physical	COM-B Model
How would you explain any planning that was required to use the system?	Capability-Cognitive	
Would there be any factors that will affect your ability to offer this system for your patient in your current work environment?	Opportunity- Physical	
How did the use of smart blister pack affect your interaction with your patients?<br>How did using the new smart bluster pack for your patients affected your interaction with your pharmacy staff and superiors i.e. managers?	Opportunity- Social	

What do you see as advantages/disadvantages of using the smart blister pack by your patient?<br>What did you see as advantage/disadvantage of access to medication adherence information for your patients?<br>Did it become easier after you start doing it?<br>If you have to do this how would you plan your workload and workflow around this?<br>How would you explain any support you received from organization if any?<br>How did you feel when you were providing the service?	Motivation	
How did you incorporate this adherence data into your practice? Was it useful and How?<br>On average how often did you check each patient's portal after giving them their blister pack?<br>On average how long did it take to set up a patient with a new blister pack? Please compare this to your process for your regular non-smart blister packs.<br>What was the longest time you spent looking at a single patient's profile and why?<br>What was the shortest time you spend looking at a single patient's profile and why?<br>Which patients did you check and why?	Behaviour	
What did you find most useful about the availability of real-time drug intake data and Why?<br>What did you find least useful about the availability of real-time drug intake data and Why?<br>Which features of portal did you like/dislike?<br>Which features of portal provided value to you?<br>Which features on portal provided limited to no value?<br>What did you think about the presentation of the information in the portal?	Perceived Usefulness	Technology Acceptance Model

How was your experience with this blister back?<br>How did you like dispensing the medications in smart product?<br>Please describe any problems you experienced with the product? How did you resolve those problems?<br>Did you use the reminder function for yourself in addition to the patient and how did you find it?<br>What did you hear back from your patients regarding the reminder?<br>How did you resolve any patient concerns related to the reminder function?	Perceived ease of use	
Would you recommend this product for your patients in the future? Why or Why not?	Intention to use	
Would there be any barriers to offer this system for your patient?	External factor	
